# Fatigue Life Prediction of TC4 Titanium Alloy Bolted Structures in Thermal Environments Below 400 °C Using an Enhanced DFR Method

**DOI:** 10.3390/ma19061210

**Published:** 2026-03-19

**Authors:** Hang Peng, Bintuan Wang, Jianbo Qin, Shiyu Li, Yan Zhou, Shancheng Cao

**Affiliations:** 1 AVIC The First Aircraft Institute, Xi’an 710089, China; penghang220530@163.com (H.P.); qinjianbo217@sina.com (J.Q.); lsy2311@sina.com (S.L.); zyadbzyadb@163.com (Y.Z.); 2 School of Astronautics, Northwestern Polytechnical University, Xi’an 710072, China

**Keywords:** fatigue life, detail fatigue rating, bolted structures, fatigue testing

## Abstract

TC4 titanium alloy bolted structures are extensively utilized in aerospace engineering, particularly within the heat-affected zones of aircraft engines. However, current studies have predominantly focused on fatigue fracture of titanium alloys at temperatures exceeding 400 °C, leaving a gap in accurate fatigue life prediction for TC4 bolted structures subjected to moderate elevated temperatures up to 400 °C. To address this limitation, this study proposes an enhanced detail fatigue rating (DFR) method that is applicable to fatigue life prediction of TC4 bolted structures under thermal environments not exceeding 400 °C. Firstly, fatigue life data were acquired from base material specimens of TC4 titanium alloy tested at 20 °C, 200 °C, and 400 °C. Secondly, an enhanced DFR method that considered the temperature-dependent thermal influence was established based on the experimental results. The enhanced DFR approach was then applied to predict the fatigue life of double-shear TC4 bolted structures, and the results were compared with those obtained via the conventional DFR method. The findings demonstrate that the enhanced DFR method improves the average fatigue life estimation accuracy by 9.29% over the conventional DFR method within the 20~400 °C range. This establishes the proposed model as a highly promising tool for evaluating the fatigue performance of TC4 bolted structures under elevated thermal conditions below 400 °C.

## 1. Introduction

With the continuous improvement in aerodynamic performance and engine efficiency, airframe structures—particularly those located within the engine’s heat-affected zone—are frequently exposed to complex service environments [1,2,3]. These components are required to sustain mechanical loads while enduring elevated temperatures, which can reach up to 400 °C. Under such thermal–mechanical coupling conditions, the fatigue properties of materials can degrade significantly compared to those at room temperature, thereby posing a severe threat to flight safety [4,5]. As high-performance structural materials, titanium alloys possess high yield strength and excellent heat resistance, offering significant advantages in such demanding applications [6,7]. Among them, TC4 (Ti-6Al-4V, designated as Grade 5 in the US standards) is a representative titanium alloy, which is widely applied in aircraft engine fans, compressor disks and other critical load-bearing airframe structures [8,9].

TC4 titanium alloy components in aircraft typically experience complex alternating loads, making fatigue their primary mode of failure [10,11]. In modern aircraft design, certain critical TC4 alloy components must operate for extended periods in coupled thermo-mechanical environments below 400 °C. Previous studies have demonstrated that elevated temperatures cause significant degradation in mechanical properties of TC4 titanium alloy, such as strength and fracture toughness, thereby substantially reducing their fatigue life [12,13,14]. Consequently, in-depth investigations into the fatigue behavior and failure mechanisms of TC4 alloys under thermal conditions have emerged as a critical research focus. However, current research on high-temperature fatigue failure of titanium alloys primarily concentrates on structures such as rockets, missiles and aeroengine casings. These components typically operate in environments exceeding 400 °C, where the fatigue failure mechanism is governed by strain fatigue [15,16].

Regarding the fatigue behavior of TC4 titanium alloy in thermal environment, Tokaji et al. conducted fatigue tests on TC4 titanium alloy at elevated temperatures of 350 °C and 450 °C, demonstrating a significant reduction in fatigue life as the temperature increased. Furthermore, fracture surface analysis indicated a marked increase in secondary crack density and accelerated crack propagation rates when the temperature rose from 350 °C to 450 °C [17]. Similarly, Wang et al. investigated the growth behavior of small cracks and established a method to predict the fatigue life of TC4 at various temperatures below 300 °C [18]. However, the current literature exhibits a notable scarcity of systematic experimental data and reliable prediction models for the specific thermal regime from 20 °C to 400 °C, necessitating in-depth research to bridge the gap between material characterization and engineering applications for TC4 structures located within engine heat-affected zones.

Furthermore, in aircraft airframe design, bolted connections remain a prevalent solution for joining structural components due to their ease of disassembly, mature manufacturing processes, and reliable mechanical properties. Moreover, the integration of symmetrical double-shear structures with titanium alloys represents a classic engineering solution, achieving an optimal balance between lightweight construction and high reliability in modern aircraft. However, the diverse and complex configurations of bolted connections make it challenging to comprehensively determine their temperature-dependent fatigue life patterns solely through experimental methods. Therefore, systematically investigating the evolution of TC4 fatigue properties at various temperatures and understanding its degradation characteristics in thermal environments are essential. Establishing a thermal fatigue life prediction model applicable to TC4 bolted joints holds critical significance for advancing the widespread application of this alloy in heat-resistant aircraft structures.

The Walker strain–life model and the Manson–Coffin method are widely utilized for predicting the high-temperature fatigue life of metallic materials [19,20]. By incorporating structural strain parameters and specific material constants, these models establish strain–life curves that accurately capture fatigue behavior under varying strain amplitudes. Furthermore, they characterize the temperature dependence of fatigue life, providing a robust theoretical framework for evaluating metallic structures in thermal environments. However, the practical implementation of these methods necessitates finite element analysis (FEA) to extract local strain parameters. For complex configurations such as aerospace bolted joints, the prohibitive computational cost associated with detailed FEA significantly restricts the engineering applicability of these strain-based models for thermal fatigue assessment.

The detail fatigue rating (DFR) method quantitatively evaluates the fatigue life of complex structures by integrating material properties, stress concentration effects, and environmental factors, making it highly suitable for fatigue analysis of bolted structures [21]. Unlike the aforementioned high-temperature fatigue life prediction models, the DFR method circumvents the need for finite element analysis. This enables the rapid assessment of structural fatigue performance during the design phase, thereby providing reference for structural optimization. While applicable to medium-to-high-temperature environments below 400 °C, the DFR calculations are highly dependent on empirical fatigue data. Specifically, fatigue tests conducted at different temperatures yield corresponding DFR values, and the thermal influence coefficient is determined based on the ratio of DFR values at room temperature and elevated temperature [22,23]. However, a major limitation of this approach is its failure to explicitly account for the temperature-induced degradation of intrinsic material properties, as it relies exclusively on macroscopic experimental results to define the thermal influence coefficient. This empirical reliance compromises its predictive reliability in thermal environments and restricts its broader engineering applicability. Therefore, improving the DFR method based on material property degradation patterns is crucial for advancing its effective application in assessing the fatigue life of aircraft heat-resistant structures [24].

In summary, aiming to accurately predict the fatigue life of TC4 bolted structures subjected to medium–high temperatures below 400 °C, an enhanced DFR method tailored for thermal conditions is proposed. The primary contribution of this work lies in the precise determination of the temperature-dependent parameters within the DFR framework. Furthermore, the finite element analysis and fracture morphology analysis are conducted to identify the fatigue-prone regions and elucidate the underlying failure mechanisms. Finally, a comparative study between the conventional DFR method and the enhanced DFR method is presented to validate the feasibility and effectiveness of the enhanced DFR method.

## 2. Fatigue Life Prediction Theory and Its Improvement

The Manson–Coffin model is widely adopted due to its explicit reliance on plastic strain as the governing factor for fatigue life. To consider the mean stress effect for the Manson–Coffin model, various modified forms have been proposed, among which the Walker strain–life prediction model is a representative one [25,26]. However, the application of these models necessitates the extraction of local stress or strain data from the structure [27,28]. Normally, the finite element modeling is relatively complex and time-consuming for evaluating the strain data. This inherent inefficiency significantly restricts the widespread adoption of such strain-based methods in engineering practice, particularly for complex configurations like bolted joints.

To address these limitations, the DFR method is frequently employed to evaluate the fatigue life of joint aircraft structures [29,30]. This approach offers computational simplicity and high predictive accuracy, thereby significantly streamlining the fatigue analysis procedure. The DFR is defined as the maximum allowable stress under a stress ratio *R* = 0.06 that enables the structure to achieve a fatigue life of 10^5^ cycles with 95% confidence and 95% reliability. Consequently, this metric represents an inherent fatigue performance parameter of the structure, independent of service-loading conditions.

### 2.1. Conventional DFR Method

The formulation of the DFR method relies on two fundamental assumptions: (1) under a constant mean stress, the *S*-*N* curve exhibits a linear relationship on the double-logarithmic coordinate system of stress amplitude versus fatigue life; (2) the equal-life curves form a family of straight lines, all intersecting the horizontal axis at *σ_m_*_0_, which represents the material’s ultimate strength. Furthermore, fatigue damage accumulation adheres to the Miner’s linear damage accumulation principle [31]. The applicability of these assumptions to TC4 titanium alloy at temperatures up to 400 °C will be validated in Section 4.

For the DFR method, it is assumed that the distribution of structural fatigue life conforms to a two-parameter Weibull distribution:

(1) $$F(N)=1-\exp[-{\left(\frac{N}{\beta }\right)}^{\alpha }]$$

in which *α* denotes the shape parameter and *β* indicates the characteristic lifetime.

The 95% fatigue life refers to the fatigue life with 95% confidence and 95% reliability, abbreviated as *N*_95/95_. *N*_95/95_ is obtained through fatigue experiments or engineering experience and its calculation formula is

(2) $$N_{95/95}=\frac{\beta }{S_{T}S_{R}S_{C}}$$

in which *S*_T_ represents the specimen coefficient, *S*_R_ signifies the reliability coefficient, and *S*_C_ indicates the confidence coefficient. The characteristic lifetime *β* is estimated by

(3) $$\hat{\beta }={\left[\frac{1}{n}\sum_{i=1}^{n}{N_{i}}^{\alpha }\right]}^{\frac{1}{\alpha }}$$

where *N_i_* denotes the fatigue life of the test specimen and *α* is normally set as 3 for titanium alloys. The DFR is evaluated as follows:

(4) $$DFR=\frac{\sigma_{m0}(1-R)}{0.94\frac{\sigma_{m0}}{\sigma_{\max}}X-(0.47X-0.53)-R(0.47X+0.53)}$$

(5) $$X=S^{\left(5-lgN_{95/95}\right)}$$

in which *R* represents the stress ratio and is set as 0.06, *σ*_max_ denotes the peak stress, *S* indicates the slope of the *S*-*N* curve, and *σ_m_*_0_ signifies the failure stress at zero stress amplitude. For titanium alloys, *S* is 2, and *σ_m_*_0_ is taken as 620 MPa [22].

The ratio of DFR values under thermal conditions to that at room temperature defines the thermal influence coefficient *η*, which is utilized to predict the structural fatigue life in thermal environment. Current methods for calculating structural DFR values under thermal conditions typically assume *σ_m_*_0_ = 620 MPa. However, this assumption is physically unjustified for high-temperature applications. Research indicates that both the yield strength and ultimate strength of TC4 titanium alloy degrade significantly as temperature increases. As material properties progressively degrade under thermal conditions, the *σ_m_*_0_ of TC4 titanium alloy also decreases accordingly. Therefore, accurately obtaining the *σ_m_*_0_ values of TC4 titanium alloy at various temperatures is a critical step for evaluating the fatigue performance of TC4 titanium alloy under thermal conditions.

### 2.2. Enhanced DFR Method

**Figure 1:**
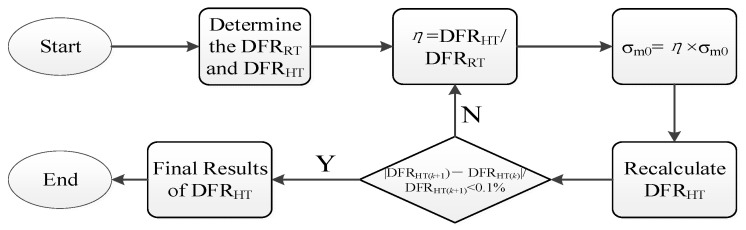
The detailed steps of the enhanced DFR method.

To address the issue of accurately evaluating the DFR for bolted joint structures under thermal environment, this paper proposes an enhanced DFR method based on the conventional DFR method while considering the influence of thermal environment on σ_m0_. The detailed procedure, illustrated in Figure 1, is outlined as follows:

(1) Based on the conventional DFR method, calculate the DFR value of the material under room temperature of 20 °C (defined as DFR_RT_) and under thermal conditions (defined as DFR_HT_).
(2) Define the thermal influence coefficient *η* = DFR_HT_/DFR_RT_.
(3) Use the structural thermal influence coefficient *η* to correct *σ_m_*_0_, *σ_m_*_0_ = *η* × *σ_m_*_0_. Substitute the corrected *σ_m_*_0_ into Equation (4) to recalculate the DFR_HT_ value and the corresponding *η*.
(4) Repeat the above steps until *σ_m_*_0_ converges, with the convergence criterion being

$$\frac{\left|{\mathrm{DFR}}_{\mathrm{HT}}(k+1)-{\mathrm{DFR}}_{\mathrm{HT}}(k)\right|}{{\mathrm{DFR}}_{\mathrm{HT}}(k+1)}<0.1\%$$

To validate the feasibility of the proposed method for calculating DFR values under thermal conditions, fatigue experiments were conducted on both base material specimens and bolted joint specimens of TC4 titanium alloy at 20 °C, 200 °C, and 400 °C, yielding corresponding fatigue life data. Based on the experimental results from the base material specimens, both the conventional DFR method and the enhanced DFR method were used to calculate the DFR values and thermal influence coefficients of TC4 titanium alloy at various temperatures. Subsequently, the thermal influence coefficients obtained from both methods were employed to predict the fatigue life of the bolted joint specimens with respect to test temperature. Finally, by comparing with the fatigue experimental results of the bolted plate specimens, the accuracy of both methods in characterizing the temperature-dependent fatigue life behavior of bolted joint structures was evaluated, thereby validating the effectiveness and accuracy of the enhanced DFR method.

## 3. Experimental Validation of the Enhanced DFR Method

In this study, systematic fatigue experiments were conducted on TC4 titanium alloy base material and bolted joint specimens to determine their fatigue lives and dispersion characteristics at various temperatures. Furthermore, finite element analysis and fatigue fracture analysis were integrated to identify the critical structural locations.

### 3.1. Experimental Introduction

**Figure 2:**
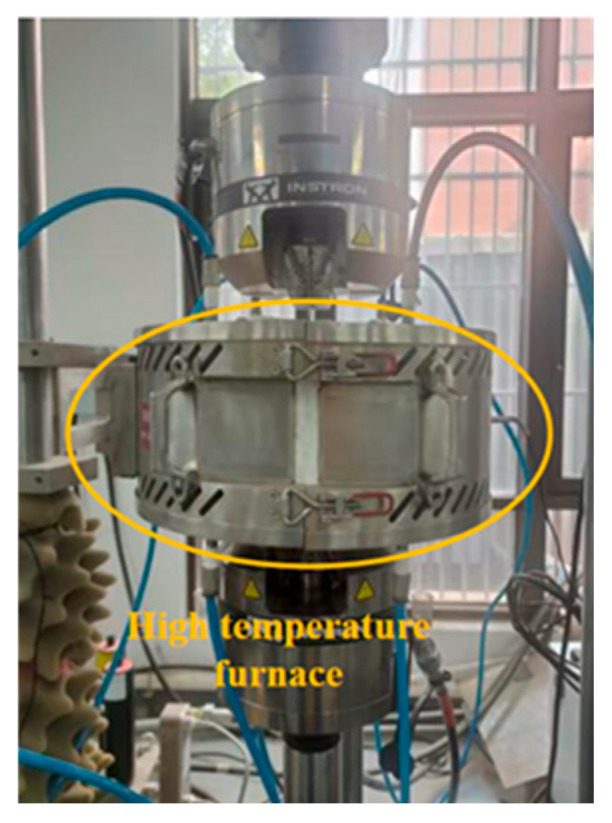
Fatigue testing based on Instron 8801.

**Figure 3:**
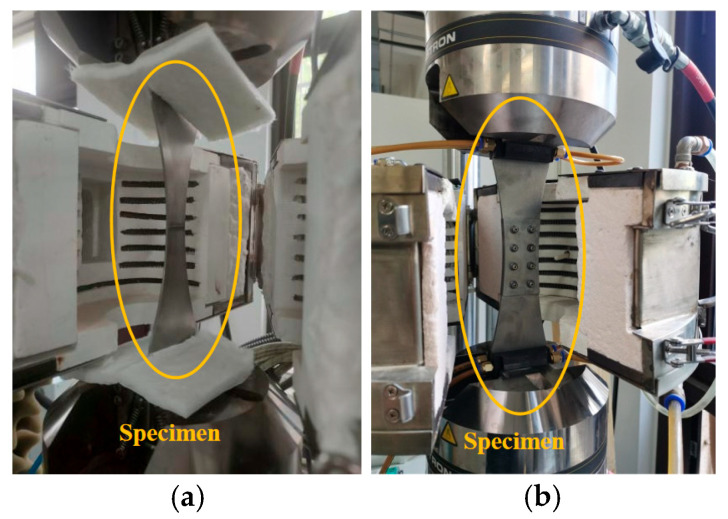
Experimental setup of (**a**) base material specimen and (**b**) bolted joint specimen.

Fatigue experiments were conducted using an Instron 8801 (Norwood, MA, USA) fatigue testing machine, with the experimental setup and loading method shown in Figure 2 and Figure 3. A sinusoidal load spectrum was applied with the stress ratio *R* = 0.06 and the loading frequency of 12Hz. For the base material specimens, the fatigue peak load was 30 kN (corresponding to a nominal stress of 600 MPa at the narrowest cross-section). For the bolted joint specimens, the fatigue peak load was 24 kN (corresponding to a nominal stress of 300 MPa at the narrowest cross-section). Specifically, a total of 48 specimens were analyzed, comprising 24 base metal specimens and 24 bolted joint specimens. For each type, eight specimens were tested at each temperature level.

**Figure 4:**
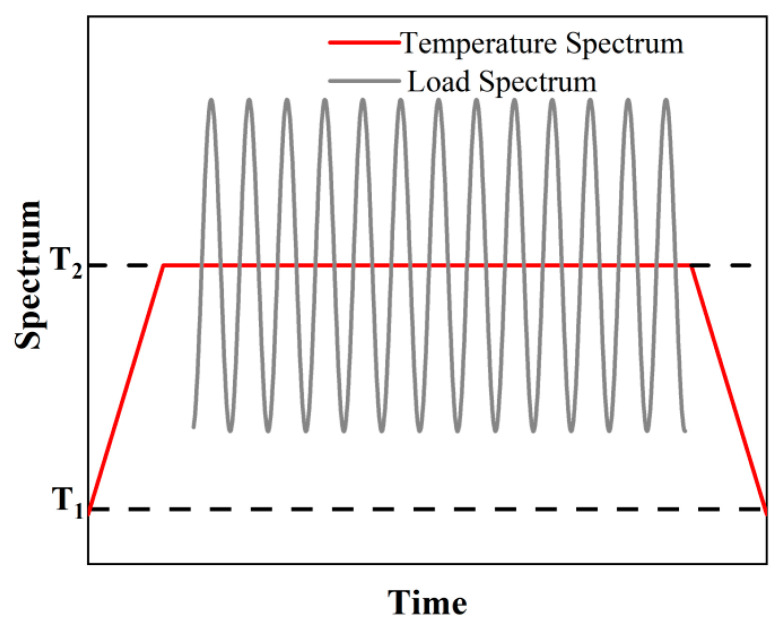
Experimental temperature spectrum and load spectrum.

The temperature loading sequence comprised three stages: a 30 min heating phase, a 30 min isothermal holding period at the target temperature during which fatigue testing was conducted until failure, and a subsequent 30 min cooling phase. Figure 4 illustrates the temperature and load spectra, with T_1_ representing room temperature and T_2_ the target temperature.

**Figure 5:**
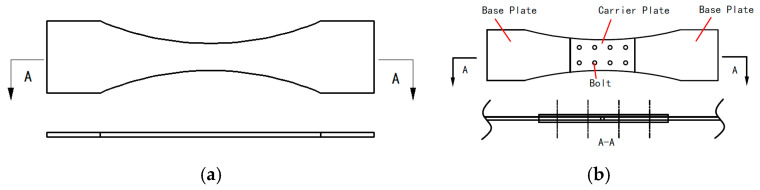
Configurations of (**a**) base material specimen and (**b**) bolted joint specimen with a double-shear configuration. ‘A’ represents cross-section view.

Fatigue tests were conducted on test specimens comprising base material and bolted connections. The material used for the “base material” specimens is completely identical to the material of the base plates and carrier plates used in the bolted joint specimens. The geometric dimensions of the test specimens are tabulated in Table 1. Flat-head titanium alloy bolts with a diameter of 5 mm were used for connection, with both bolt spacing and row spacing set at 20 mm. The bolt is a deformable TC4 component with a friction coefficient of 0.1 and a preload of 5000 N. The structural schematic of the test specimens is shown in Figure 5. Both types of test specimens were fabricated from TC4 titanium alloy. Their dimensions were designed according to the specific form of the application object and its connection characteristics.

### 3.2. Finite Element Analysis for Fatigue Zones

To identify potential fatigue zones in the specimens and provide a basis for excluding test data with abnormal fracture locations in subsequent fatigue tests, this section conducted finite element analysis on the two types of specimens. Based on the geometric parameters of the specimens given in Table 2, corresponding finite element models were established. Moreover, material parameters of TC4 titanium alloy at different temperatures are listed in Table 2.

**Figure 6:**
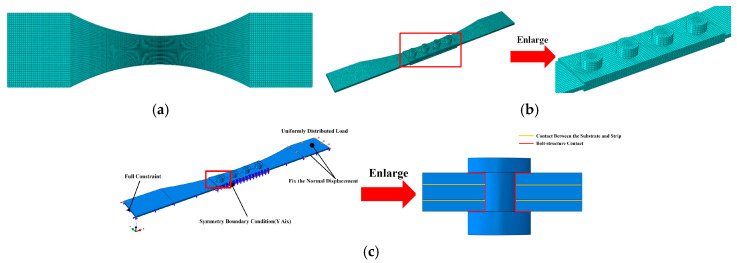
(**a**) FE model of base material specimen; (**b**) FE model of bolted joint specimen; (**c**) boundary conditions and bolt contact configuration for bolted joint specimen.

Based on the stress characteristics of the specimens, one end is fixed while a uniformly distributed load is applied to the other end (for the base material test specimen, the peak stress of 600 MPa at the narrowest point in the middle is used for conversion; for bolted connection specimens, the calculation is based on 300 MPa at the narrowest point of the substrate). To reduce computational load, a 1/2 model was established for the bolted connection specimen based on its structural form and stress characteristics. Each bolt was modeled as a solid element, with contact defined between the bolt, substrate, and flange plate. The contact type is friction contact with a friction coefficient of 0.1, and the normal contact type is hard contact. A preload of 5000 N was applied to the bolts. The modeling approach for the bolted connection structure is illustrated in Figure 6c.

**Figure 7:**
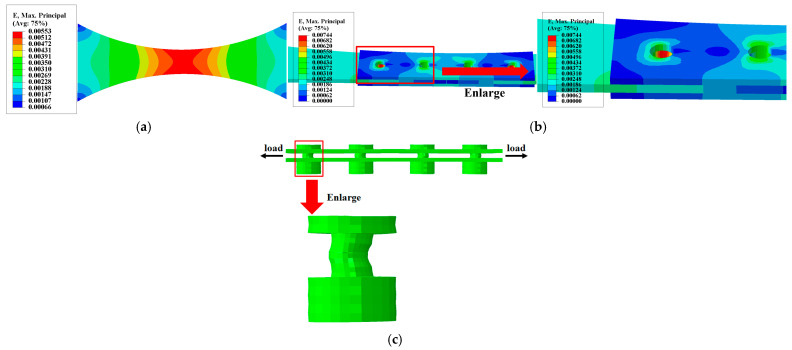
Maximum principal strain distribution at 20 °C of (**a**) base material specimen and (**b**) bolted joint specimen; (**c**) deformation diagram of the bolt (magnified 60 times).

The model employs hexahedral eight-node reduced integration elements (C3D8R). For bolted structures, bolts share mesh nodes with base plates and flange plates to facilitate model convergence, with local refinement applied at critical locations as shown in Figure 6. The finite element analysis results for the two types of specimens at 20 °C are presented in Figure 7. Under thermal conditions, the strain distribution patterns for both specimen types remain largely consistent with those at 20 °C, though numerical values differ. It indicates that the maximum principal strain in the base material specimen is concentrated at the minimum cross-section near the specimen center, while the maximum principal strain in the bolted joint specimen occurs near the first row of bolt holes at the substrate end. Under tensile testing, cracks tend to initiate first in the maximum principal strain zone, subsequently propagating outward along the stress gradient, ultimately leading to specimen failure.

Figure 7c displays the deformation contour map of the fastener in the bolted joint test specimen. The figure reveals that the fastener bulges toward the loaded side at the base plate, while its deformation direction at the flange plate opposes that of the base plate, exhibiting an overall “*S*”-shaped geometric deformation characteristic. Furthermore, “hard contact” exists between the fastener head and nut with the flange plate, restricting the fastener’s free deformation in the vertical direction.

### 3.3. Fatigue Fracture Analysis

**Figure 8:**
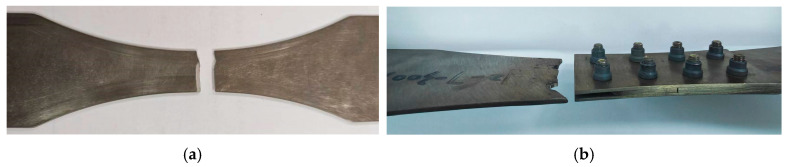
Fatigue failure modes of (**a**) base material specimen and (**b**) bolted joint specimen.

The fracture morphology of those two types of specimens is presented in Figure 8. By observing the macroscopic fracture surface characteristics of each specimen group, distinct fatigue source locations can be clearly identified. Fracture in the base material specimens primarily occurs at the minimum cross-sectional area, while fracture in the bolted joint specimens is mainly concentrated near the first row of bolt holes at the substrate end. The fracture locations highly align with the fatigue hazard zones of finite element analysis. The fatigue failure process involves three main stages: crack initiation, crack propagation, and fatigue fracture [32].

**Figure 9:**
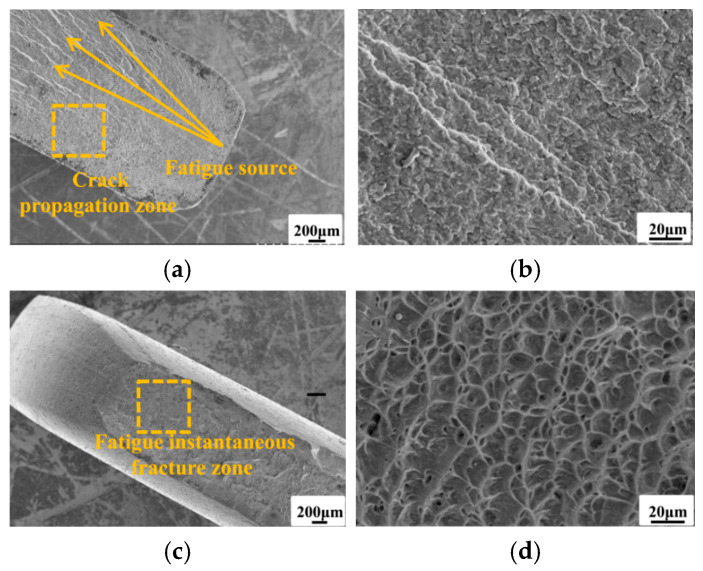
Typical fracture morphologies of base material specimen (**a**) crack initiation zone; (**b**) crack propagation zone; (**c**) fatigue instantaneous fracture zone; (**d**) ductile dimple morphology.

To further analyze the fatigue failure mechanism of the structure, fracture surfaces from representative failed specimens were examined by scanning electron microscope. Fracture surface examination serves as a means to validate the validity of fatigue test results. It forms the foundation for subsequent application or refinement of the DFR method, with fracture analysis of anomalous data being particularly crucial. Additionally, fracture surfaces can be compared with finite element results to assess whether crack initiation locations align, thereby verifying the accuracy of finite element analysis. Figure 9a shows the microstructure at the crack initiation site of the base metal specimen and Figure 9b presents a magnified view of the crack propagation zone from Figure 9a. Figure 9c displays the microstructure of the fatigue instantaneous fracture zone, exhibiting ductile dimple characteristics with low surface flatness. Figure 9d provides a close-up of the ductile dimples in Figure 9c, revealing uniformly sized, small yet deep dimples. Localized areas feature flat, adhesive-like surfaces, indicating ductile fracture behavior.

**Figure 10:**
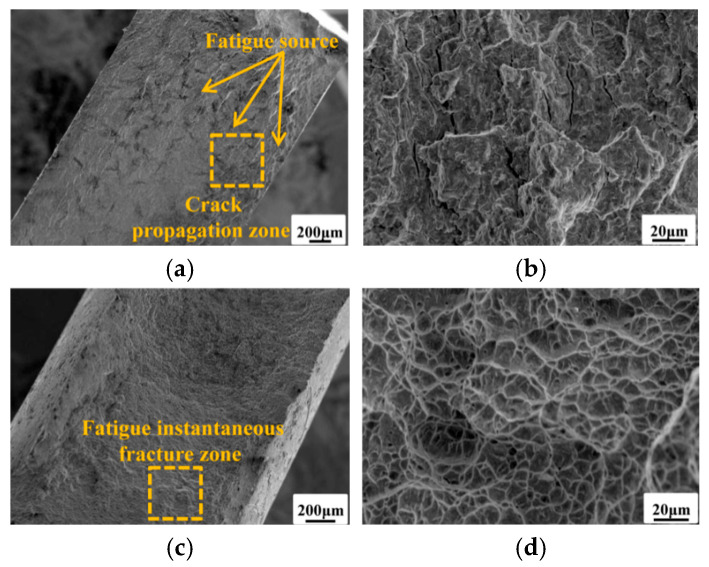
Typical fracture morphologies of bolted joint specimen (**a**) crack initiation zone; (**b**) crack propagation zone; (**c**) fatigue instantaneous fracture zone; (**d**) ductile dimple morphology.

For bolted joint specimens, crack initiation sites occur at defects within the stress concentration zone around bolt holes as shown in Figure 10. The microstructural features of both the crack propagation zone and fatigue instantaneous fracture zone resemble those of the base metal specimens, exhibiting pronounced ductile fracture characteristics.

It is important to note that the macroscopic fatigue life and the parameters used in the enhanced DFR method are fundamentally governed by the microstructure of the TC4 alloy. At temperatures below 400 °C, the reduction in the service life of the TC4 titanium alloy is not caused by phase transformation. Instead, it occurs because thermal energy diminishes the material’s resistance to micro-deformation leading to a decrease in the material’s ultimate strength *σ_m_*_0_. While the current study primarily focuses on a macroscopic phenomenological prediction model for engineering structures, the inherent microstructural degradation at elevated temperatures is the physical root cause of the decline in the DFR parameters. Future work will focus on coupling microstructural damage mechanisms with the macroscopic DFR method to provide a more comprehensive multi-scale life prediction framework.

### 3.4. Experimental Results

After experiments, outlier fatigue life values are removed according to Chauvenet’s criterion. The experimental results are tabulated in Table 3.

As shown in Table 3, the fatigue life ranges from 1.8 × 10^4^ to 1.9 × 10^5^ cycles for the base material specimens, while the fatigue life ranges from 4.4 × 10^4^ to 2.7 × 10^5^ cycles for the bolted joint specimens. It indicates that as the experimental temperature increased from 20 °C to 400 °C, the mean fatigue life and dispersion of both specimen types show a decreasing trend, reflecting the adverse effect of thermal environment on fatigue performance. In addition, within the temperature range from 20 °C to 400 °C, both types of specimens exhibited stress fatigue failure behavior.

## 4. Comparison Study of the Enhanced DFR Method

In this section, the enhanced DFR method is compared with the conventional DFR method to validate its accuracy and effectiveness. Firstly, the thermal influence coefficient is estimated for different temperatures based on the base material specimen. And then, the evaluated thermal influence coefficients are adopted to predict the fatigue life of bolted joint specimens under thermal conditions. Finally, in comparison with the experimental fatigue life of bolted joint specimens, the higher accuracy of the enhanced DFR method is demonstrated.

### 4.1. Estimation of Thermal Influence Coefficient

To facilitate explanation of the calculation process, the DFR method is detailed using the base material specimen at 20 °C as an example. Based on the experimental results, the characteristic life *β* for base material specimens at 20 °C is calculated as

(6) $$\hat{\beta }={\left[\frac{1}{7}\sum_{i=1}^{7}{N_{i}}^{3}\right]}^{\frac{1}{3}}=122648$$

Normally, the test specimen coefficient *S*_T_ is set as 1.3, the reliability coefficient *S*_R_ is 2.7, and the confidence coefficient *S*_C_ is 1.18. Therefore, the formula for calculating the test specimen’s double 95% life *N*_95/95_ is

(7) $$N_{95/95}=\frac{122648}{1.3\times 2.7\times 1.18}=29612$$

Calculations based on Equations (4) and (5) yield a DFR value of 493.62 MPa for the base material specimens at 20 °C. This signifies that under a stress ratio *R* = 0.06 and a stress level of 493.62 MPa, the fatigue life of the test specimen achieves 10^5^ cycles with 95% confidence and 95% reliability.

Correspondingly, the *N*_95/95_ lifetimes of TC4 titanium alloy at 200 °C and 400 °C are 15,650 and 5751 cycles, respectively, with DFR values of 439.81 MPa and 360.66 MPa. Using the DFR value of specimens tested at 20 °C as the baseline, the ratio of the DFR value of specimens tested under thermal conditions to that at 20 °C is *η*. This coefficient characterizes the attenuation effect of thermal environment on material fatigue performance. Its calculation formula is

(8) $$DFR_{\mathrm{HT}}=DFR_{\mathrm{RT}}\times \eta$$

**Figure 11:**
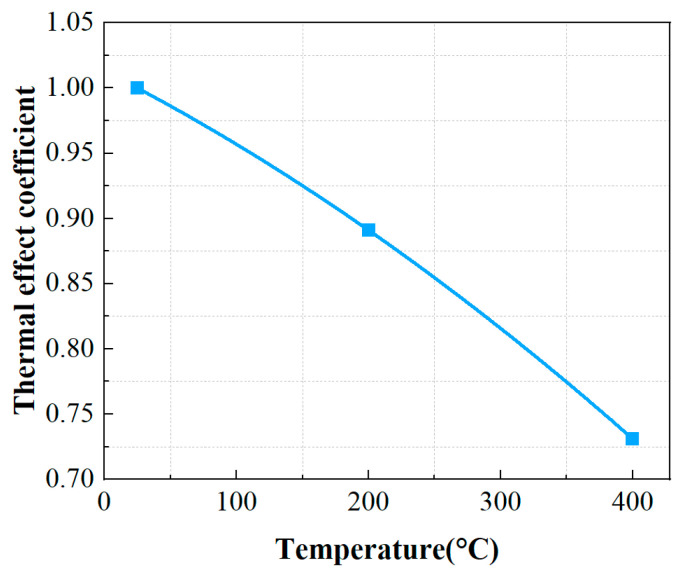
Thermal influence coefficient *η* at different temperatures of the conventional DFR method.

The *η* coefficients for TC4 titanium alloy base material specimens are 0.89 at 200 °C and 0.73 at 400 °C, respectively. Figure 11 illustrates the decrease pattern of thermal influence coefficient *η* based on base material specimens by increasing temperature.

**Figure 12:**
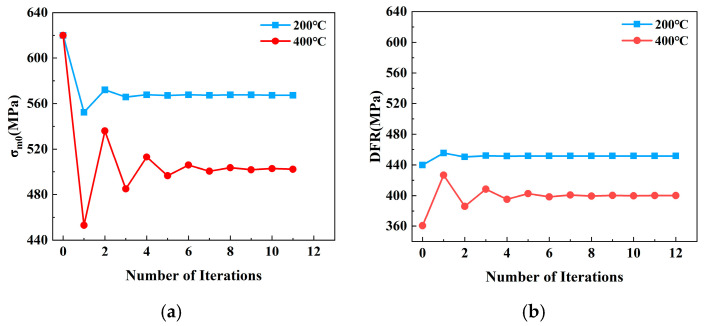
The enhanced DFR method of base metal specimen: (**a**) the corrected *σ_m_*_0_ values; (**b**) the corrected DFR values.

The DFR values of the base metal specimens were obtained using the conventional DFR method at different temperatures. Then, the DFR and *σ_m_*_0_ values were iteratively calculated based on the proposed DFR method as illustrated in Figure 1. Figure 12 displays the iterative results of *σ_m_*_0_ and DFR values for the test specimens at 200 °C and 400 °C. The results indicate that as the iteration count increases, the *σ_m_*_0_ and DFR values of the test specimens eventually converge to stable values. Specifically, at 200 °C, the *σ_m_*_0_ value of the test specimen is 567.73 MPa, with a corresponding DFR value of 451.50 MPa. At 400 °C, the final *σ_m_*_0_ value reached 502.77 MPa, with a corresponding DFR value of 399.82 MPa.

**Figure 13:**
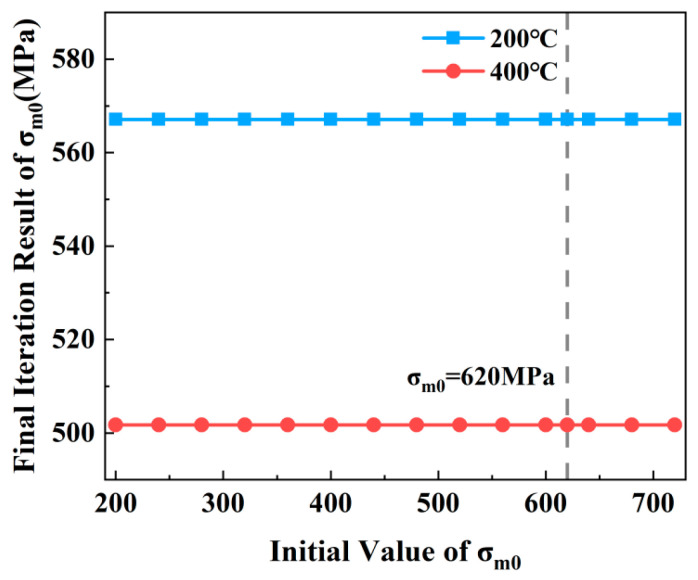
Final corrected *σ_m_*_0_ values for different initial *σ_m_*_0_ values.

Figure 13 shows the final *σ_m_*_0_ correction results based on an initial *σ_m_*_0_ value of 620 MPa. In reality, the *σ_m_*_0_ value of TC4 titanium alloy should decrease below 620 MPa as temperature increases. Figure 13 further illustrates the final iteration results corresponding to different initial *σ_m_*_0_ values. The results indicate that within a reasonable range, different initial *σ_m_*_0_ values have negligible impact on the final iteration outcome. This implies that despite variations in the initial *σ_m_*_0_ value, the final iterated *σ_m_*_0_ converges to the same stable value. Correspondingly, the final DFR value of the structure also converges to the same result.

**Figure 14:**
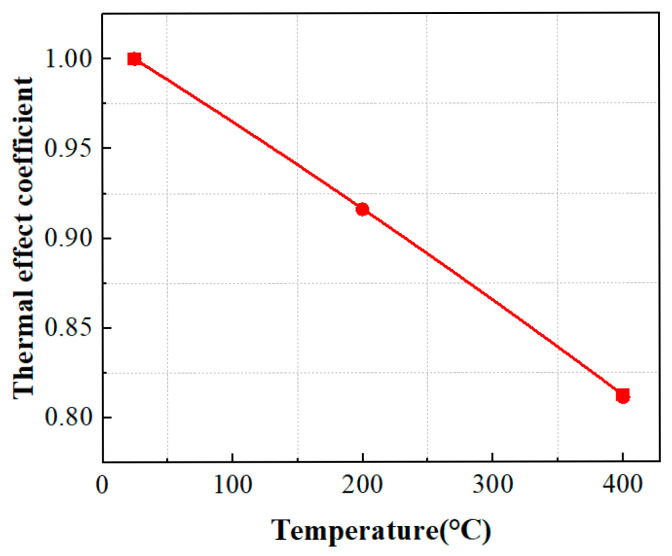
Thermal influence coefficient at various temperatures of the proposed DFR method.

Based on the DFR and *σ_m_*_0_ values of TC4 titanium alloy specimens at 20 °C, the thermal influence coefficients are defined for materials at 200 °C and 400 °C, respectively. These coefficients are determined by the ratio of corresponding DFR and *σ_m_*_0_ values under thermal conditions to those at room temperature. The decay ratio of the material’s DFR value under thermal conditions is identical to that of *σ_m_*_0_. At 200 °C and 400 °C, the thermal influence coefficients of the material correspond to 0.92 and 0.81, respectively. As shown in Figure 14, the thermal influence coefficient *η* exhibits a pronounced decreasing trend with increasing test temperature. Compared to the conventional DFR method in Figure 11, the differences in the thermal influence coefficients are significant.

### 4.2. Validation of the Enhanced DFR Method

Based on base material specimens, the thermal influence coefficient *η* are evaluated for the conventional DFR method and the enhanced DFR method under thermal conditions. To validate the effectiveness of the enhanced DFR method, fatigue life prediction errors from both methods were compared and analyzed using fatigue test results of bolted joint specimens. The major steps are summarized as follows:

(1) Using the DFR value of the bolted plate specimen at 20 °C as the reference, based on the evaluated coefficient *η* as shown in Figure 11 and Figure 14, compute the DFR values for the bolted joint specimen under thermal conditions;
(2) Based on the predicted DFR values under thermal conditions for the bolted joint specimens, the fatigue life values of the bolted joint specimens at various temperatures were calculated;
(3) Compare the errors between the fatigue life values predicted by the two methods and the experimental values of the bolted joint specimens.

The thermal influence coefficient *η* obtained through conventional DFR methods and the enhanced DFR calculation method proposed in this paper was used to correct the DFR values and *σ_m_*_0_ values of the TC4 bolted joint specimens. The results are shown in Table 4.

Based on the DFR values of bolted joint specimens at 20 °C, the DFR and *σ_m_*_0_ values for bolted joint specimens at elevated temperatures are calculated. The structural fatigue life is then predicted using the following fatigue life calculation formula:

(9) $$Z=\frac{\sigma_{\max}(1-R)(\sigma_{m0}-0.53DFR)}{DFR[0.94\sigma_{m0}-0.47(1+R)\sigma_{\max}]}$$

(10) $$N_{95/95}=10^{\left(5-\frac{\mathrm{lg}Z}{\mathrm{lg}S}\right)}$$

The *N*_95/95_ life calculation method is given by Equations (9) and (10). Table 5 presents the prediction results from both approaches. Across the temperature range of 20 °C to 400 °C, the proposed DFR calculation method demonstrated superior accuracy in predicting fatigue life at elevated temperatures.

**Figure 15:**
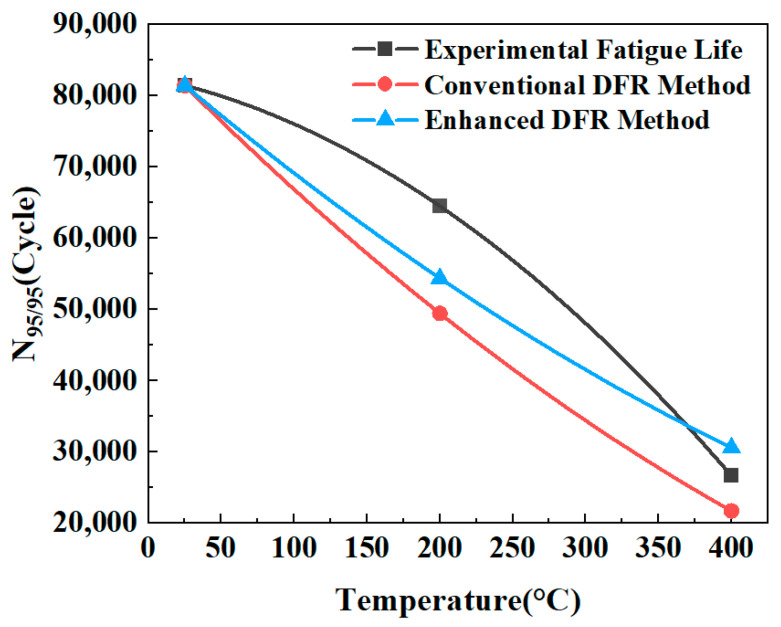
Experimental and predicted fatigue life results of bolted joint specimens.

Figure 15 displays the fatigue life prediction results for bolted joint specimens across the temperature range from 20 °C to 400 °C, based on the conventional DFR Method and the enhanced DFR method. As shown in Figure 15, the mean relative error (MRE) in the range of 20 °C to 400 °C is calculated as

(11) $$MRE=\frac{1}{T_{2}-T_{1}}\times \int_{T_{1}}^{T_{2}}\left|\frac{N_{ture}(T)-N_{pred}(T)}{N_{ture}(T)}\right|dT$$

in which, *T*_1_ and *T*_2_ represents 20 °C and 400 °C, respectively.

Based on Equation (11), the MRE for the conventional DFR method and the enhanced DFR method are evaluated as 19.89% and 10.60%, respectively. It indicates that the average prediction accuracy of the enhanced DFR method is increased by 9.29% in the range of 20 °C to 400 °C when compared with the conventional method, which validates the better prediction performance of the enhanced DFR method.

## 5. Conclusions

This study systematically investigated the fatigue behavior of base material specimens and bolted joint specimens at various temperatures (20 °C, 200 °C, and 400 °C). Based on the experimental results of the base material specimen, the conventional detail fatigue rating (DFR) method was evaluated. Furthermore, an enhanced DFR method tailored for elevated temperatures is proposed and validated using the fatigue test data of the bolted joint specimens, confirming its applicability and accuracy. Two major findings are summarized as follows:

(1) In the enhanced DFR method, despite variations in the initial *σ_m_*_0_ value, the final iterated *σ_m_*_0_ converges to the same stable value. Similarly, the final DFR value of the structure also converges to the same result.
(2) The enhanced DFR method for TC4 titanium at elevated temperatures accounts for the thermal effects on ultimate strength. Compared to the conventional DFR method, it yields superior results, thereby improving the average fatigue life prediction accuracy for bolted joint specimens across the 20 °C to 400 °C range.

Furthermore, it should be noted that the factors presented in Equations (8)–(10) are not absolute constants; rather, they are highly dependent on the specific material composition, initial structure, operating atmosphere, and other experimental conditions. The superior predictive capability of the enhanced DFR method is fundamentally attributed to its solid physical and mechanical basis. Methodologically, unlike the traditional DFR approach, the improved model dynamically couples the temperature-dependent degradation of the material’s ultimate strength. Physically, this improvement captures the actual mechanical response of the TC4 alloy under elevated temperatures, where the material’s resistance to crack initiation decreases. By integrating this physical degradation mechanism into the parameter of the DFR method, the enhanced DFR method successfully bridges the gap between material-level thermal softening and structure-level fatigue life prediction, thereby reducing prediction errors.

## Figures and Tables

**Table 1:** Geometric dimensions of experimental specimens (mm).

Specimen Type	Length	Clamping End Width	Width of Center Part	Thickness of Base Plate	Thickness of Carrier Plate
Base material specimen	300	66	25	2	/
Bolted plate	300	66	40	2	1.5

**Table 2:** Material parameters of TC4 titanium alloy at different temperatures.

Temperature (°C)	20	200	400
*E* (GPa)	112	104	92
Poisson ratio *v*	0.34	0.34	0.37
*σ*_0.2_ (MPa)	990	780	627
*σ_b_* (MPa)	1060	854	730
Failure plastic strain	0.0992	0.1069	0.1119

**Table 3:** Fatigue test results for two types of experimental specimens.

Specimen Category	Temperature (°C)	Fatigue Life							Average Fatigue Life	Standard Deviation
Base material specimen	20	61,116	96,859	57,233	83,511	209,546	106,190	84,777	99,890	51,467
	200	63,735	42,939	45,067	53,692	39,726	66,755	98,713	58,661	20,456
	400	22,247	18,752	23,290	20,464	30,793	25,869	/	23,569	4293
Bolted joint specimen	20	268,197	53,722	264,828	251,418	84,360	226,355	191,175	191,436	88,059
	200	139,829	118,381	84,456	149,107	203,604	136,566	246,516	154,065	54,243
	400	90,446	52,703	74,873	52,970	44,602	81,005	/	66,099	18,471

**Table 4:** Correction results of DFR values and *σ_m_*_0_ values for bolted joint specimens.

Temperature (°C)	Conventional DFR Method		Enhanced DFR Method	
	DFR (MPa)	*σ_m_*_0_ (MPa)	DFR (MPa)	*σ_m_*_0_ (MPa)
20	286.43	620	286.43	620
200	255.21	620	262.37	567.92
400	209.38	620	232.29	502.82

**Table 5:** Prediction results of *N*_95/95_ life of bolted joint specimens.

Temperature	*N*_95/95_ Test Results	*N*_95/95_ Prediction Results (Conventional DFR)	*N*_95/95_ Prediction Results (Enhanced DFR)
200 °C	64,507	49,463	54,693
400 °C	26,663	21,786	30,742

## Data Availability

The data presented in this study are available on request from the corresponding author. The data are not publicly available due to privacy.
